# Newly Discovered Fungal Species from Black Pepper Marketed in Brazil: *Penicillium pipericola* sp. nov. and *Syncephalastrum brasiliense* sp. nov.

**DOI:** 10.3390/microorganisms13122691

**Published:** 2025-11-25

**Authors:** Vinicius S. Rosa, Adriana R. P. da Silva, Paola Ferrari, Daniela B. B. Trivella, Mariana C. de Souza, Lara Durães Sette, Rafael de Felício, Beatriz T. Iamanaka, Marta H. Taniwaki, Josué J. Silva

**Affiliations:** 1 Centro de Ciência e Qualidade de Alimentos, Instituto de Tecnologia de Alimentos, Campinas 13070-178, SP, Brazil; 2 Brazilian Biosciences National Laboratory (LNBio), Brazilian Center for Research in Energy and Materials (CNPEM), Campinas 13083-100, SP, Brazilrafael.felicio@lnbio.cnpem.br (R.d.F.); 3 Instituto de Biociências, Universidade Estadual Paulista (UNESP), Rio Claro 13506-900, SP, Brazil; lara.sette@unesp.br

**Keywords:** new species, phylogeny, spices, metabolome, mycobiota, diversity

## Abstract

Black pepper (*Piper nigrum* L.) has historically been among the most consumed spices globally. Brazil is one of the world’s largest producers and exporters, and is the largest in the Western Hemisphere. This study describes two new fungal species associated with black pepper commercialized in Brazil. The first, *Penicillium pipericola* sp. nov., belongs to the subgenus *Penicillium*, section *Paradoxa*, series *Atramentosa*. The second, *Syncephalastrum brasiliense* sp. nov., belongs to the order Mucorales, family Syncephalastraceae. The taxonomic classification of these species was supported by a pluralistic approach, based on multilocus phylogenetic analyses, morphological analyses, and metabolomics. Furthermore, the metabolomic analysis revealed considerable biosynthetic versatility of the new species under different cultivation conditions, producing metabolites with therapeutic and biotechnological potential. The identification of these species increases the understanding of fungal diversity in the black pepper production chain and may have important implications for the microbiological quality of the product, for the understanding of ecological interactions within the agroecosystem and for potential industrial applications.

## 1. Introduction

Black pepper (*Piper nigrum* L.), often referred to as the “queen of spices,” has significant economic and cultural importance as the most widely consumed spice in the world. Native to India and belonging to the Piperaceae family, this tropical plant is cultivated in several regions of the world, with Brazil standing out as the second largest producer and the main exporter in the Western Hemisphere [1,2,3]. It contains several bioactive compounds, including alkaloids, terpenoids, phenolics, and flavonoids, with piperine being a key component [4,5]. Brazilian production is concentrated in the states of Espírito Santo and Pará, which collectively account for over 90% of national output [6]. The fruits of *P. nigrum* are processed into black or white pepper, serving diverse purposes in the culinary, pharmaceutical, cosmetic, and food industries due to their essential oils, resins, and piperine content [2].

Despite its global prominence, the production and processing of black pepper face significant challenges regarding microbial contamination. During post-harvest handling, including drying, storage, and packaging, the product is exposed to environmental and handling conditions conducive to microbial growth. Fungi, particularly storage-associated genera such as *Aspergillus* and *Penicillium*, are frequent contaminants capable of altering organoleptic properties, reducing market value and, most alarmingly, producing toxic secondary metabolites known as mycotoxins [7,8]. Among these, aflatoxins and ochratoxin A (OTA) are of particular concern due to their potential carcinogenicity, immunosuppressive effects, and hepatotoxicity [9,10]. The initial diversity of fungal contamination plays a crucial role in the final quality of pepper products [11].

Recent studies have shown that spices harbor complex microbial communities dominated by *Aspergillus* and *Penicillium* species, many of which are toxigenic or opportunistic pathogens. Garcia et al. [12] found high fungal contamination in several spices, with black and white peppers exhibiting particularly high frequencies of toxigenic *Aspergillus* species, including *A. flavus* (up to 32%) and the *A. niger* complex (up to 52%), with 14.2–66.7% of *A. flavus* isolates producing aflatoxins. Jeswal & Kumar [13] identified *A. flavus* and *A. niger* as the predominant species in Indian spices, with red pepper exhibiting 85.4% aflatoxin contamination and the highest aflatoxin levels (219.6 ng/g). Mandeel [14] reported similar results in imported spices, with red pepper and black pepper exhibiting the highest contamination (1580 and 1120 CFU/g, respectively), predominantly by the genera *Aspergillus* and *Penicillium*. Black pepper is generally reported to have high fungal loads; however, mycotoxin levels are generally below regulatory limits. However, high consumption and the risk of cumulative exposure justify closer monitoring of the fungal biodiversity of this spice.

The Brazilian regulatory framework is on par with international standards by establishing strict microbiological and mycotoxin limits to ensure food safety [15,16]. However, compliance remains an ongoing challenge, as contamination is frequently reported in the production chain. This highlights the importance of interventions in agronomic practices and advanced microbial monitoring and control strategies.

In addition to contamination, the microbial diversity associated with black pepper may offer a distinct perspective for examining fungal ecology and taxonomy. Spices are an underexplored niche that contains a wide diversity of fungal taxa, including previously undescribed species.

Polyphasic taxonomy combines genotypic and phenotypic data and has contributed significantly to the delimitation of fungal species and the understanding of biodiversity. This approach integrates diverse data types, such as molecular phylogeny, morphology, physiology, and chemotaxonomic markers, into consensus classification systems [17]. In addition to taxonomy, omics approaches such as genomics, transcriptomics, proteomics, and metabolomics enable more accurate fungal identification and taxonomy, beyond traditional morphological approaches [18]. These multiomics approaches are particularly useful for mycotoxin research, as they help identify and characterize the gene clusters responsible for the production of these metabolites [19]. On the other hand, omics approaches have also facilitated the discovery of beneficial fungal secondary metabolites, including antibiotics, drugs, and other bioactive compounds, highlighting the great versatility of fungi [20].

In this study, we report two new species of fungi associated with black pepper commercialized in Brazil. The first species is named *Penicillium pipericola* sp. nov. and belongs to the subgenus *Penicillium* sect. *Paradoxa* Ser. *Atramentosa*. The genus *Penicillium* is highly diverse and globally distributed, with many species frequently isolated from soil, organic waste, and a variety of food products. Section *Paradoxa* accommodates species that are primarily isolated from plant material and are notable for their rich secondary metabolism, which may include the production of bioactive compounds [21,22,23].

The second species, *Syncephalastrum brasiliense* sp. nov., is classified within the order Mucorales, family Syncephalastraceae, genus *Syncephalastrum*. Members of the genus *Syncephalastrum* are distinguished by the formation of spores in characteristic clusters, arranged in structures resembling bunches or masses, and are commonly found in soil and decaying organic matter [24]. The occurrence of *S. brasiliense* sp. nov. in black pepper is therefore an intriguing discovery, as this genus does not belong to the typical groups of microorganisms associated with spice crops. Discovering members of this group within the black pepper production chain raises questions about their ecological role and potential interactions with other microorganisms or even with the pepper plant itself.

This study aimed to describe, based on a polyphasic approach, two new fungal species obtained from black pepper commercialized in Brazil.

## 2. Materials and Methods

### 2.1. Samples

The strains corresponding to the two candidate species (ITAL-3JA, ITAL-3JB, and ITAL-3JC of *P. pipericola* sp. nov., and ITAL-36NA, ITAL-36NB, and ITAL-36NC of *S. brasiliense* sp. nov.) were isolated from black pepper samples obtained from retail establishments in the city of Campinas, São Paulo State, Brazil. The isolation procedure followed the methodology of direct plating of grains described in Pitt and Hocking [25]. The surfaces were disinfected in a 0.4% sodium hypochlorite solution for 2 min, then 50 grains were plated on Dichloran Glycerol Agar 18% (DG18). Each sample weighed approximately 150 g.

### 2.2. Morphological Analysis

Initially, the fungi were evaluated based on their macroscopic characteristics. All fungal isolates were purified and plated at three equidistant points on Czapek Yeast Extract Agar (CYA) and Malt Extract Agar (MEA) at 25 °C and incubated for 5 days. They were subsequently morphologically characterized according to the taxonomic keys of [25,26,27], supplemented by other sources when necessary. Morphological identification was limited to the fungal genus/group (morphogroup) level and subsequently combined with molecular analysis.

For *P. pipericola* sp. nov. analysis, morphological studies were performed according to the methodology proposed by Visagie et al. [23]. To evaluate the macroscopic characteristics, strains of candidate species were plated on different media: CYA, MEA, DG18, Creatine Sucrose Agar (CREA), and Yeast Extract Sucrose Agar (YESA). Incubation was performed in the dark at 25 °C for 7 days. Additionally, strains were tested on CYA medium at 30 °C and 37 °C for 7 days. While for *S. brasiliense* sp. nov., morphological analyses were performed on MEA, Potato Dextrose Agar (PDA), and Sucrose Dextrose Chloramphenicol Agar supplemented with Gentamicin (SDCA+G) media at temperatures (5 °C, 25 °C, 40 °C, and 45 °C) over 7 days. All experiments were performed in triplicate.

For microscopic analysis, slides were prepared using lactic acid from colonies grown on MEA medium for 7 days at 25 °C. Micromorphological characteristics, including the size of conidiophores, stipes, vesicles, conidia, metula, and phialides, were measured using Carl Zeiss™ AxioVision Release 4.8.2 software. Observations were performed with a Zeiss Axio Imager. A2 (Carl Zeiss Microscopy GmbH, Jena, Germany), an upright optical microscope). Measurements and imaging were conducted under brightfield contrast at magnifications of 400× and 1000×.

### 2.3. Molecular Analysis

For genomic DNA extraction, purified strains were grown in liquid Yeast Sucrose medium (YES) at 25 °C for 3 days until mycelial membranes were formed, and then were macerated with liquid nitrogen. This material was used to obtain genomic DNA according to the manufacturer’s protocol (PureLink Mini Kit, Thermo Fisher Scientific, MA, USA). DNA was quantified using spectrophotometry (NanoDrop^®^, Thermo Fisher Scientific, MA, USA).

Different loci were used for molecular identification of fungal isolates. The best loci for each group were selected according to the literature and current taxonomic context. Amplification of the universal fungal barcode, ITS region (*rRNA*), was performed using the primer pair its1/its4 described in White et al. [28]; for amplification of Large subunit ribosomal ribonucleic acid (*LSU rRNA*), we used the primer pair D1/D2 described in Hoog et al. [29]. Part of the calmodulin gene was amplified using the primer pairs CF1/CF4 [30]. The beta-tubulin gene (*BenA*) was amplified using the primers Bt2a and Bt2b [31]. The locus of the second largest subunit of RNA polymerase II (*RPB2*) was amplified using the primers 5Feur/7CReur, described in Houbraken et al. [32]. The amplification conditions were the same as those described in Silva et al. [33].

After amplification, the PCR products were separated by agarose gel electrophoresis (1% *w*/*v*), stained with ethidium bromide, and visualized under ultraviolet light. After amplification, PCR product purification was performed using ExoSAP-IT™ PCR Product Cleanup (Thermo Fisher Scientific, Santa Clara, CA, USA). PCR fragments were then subjected to direct sequencing using the method described by Sanger et al. [34]. Sequencing was performed bidirectionally (forward and reverse) using a BigDye^®^ Terminator v3.1 Cycle Sequencing Kit (Applied Biosystems, Foster City, CA, USA) on a SeqStudio Genetic Analyzer^®^ (Applied Biosystems, Waltham, MA, USA).

Sequence alignment was performed using ClustalW in BioEdit Sequence Alignment Editor v.7.1.3.0. Concatenation of the loci was performed using DnaSP v.6 DNA Sequence Polymorphism.

Maximum likelihood (ML) trees were constructed separately for each locus and for the combined data set. Sequence alignment was performed with the type or neotype strain sequences of species formally accepted within each taxonomic group (*Penicillium* or *Syncephalastrum*) using ClustalW.

For ML tree construction, the optimal nucleotide substitution model was determined using jModelTest2 based on the Akaike information criterion (AIC). ML trees were constructed using MEGA 11, using 1000 bootstrap replicates, its final visualization took place through the ITOL platform (Interactive Tree of Life, https://itol.embl.de/itol.cgi, accessed on 11 October 2025). The GenBank accession numbers corresponding to the sequences used in the phylogenetic analysis are provided in Supplementary Material S1.

### 2.4. Secondary Metabolites Analysis

Secondary metabolites were extracted following adapted protocols from Smedsgaard [35] and Houbraken et al. [36]. Fungal strains were grown for 7 days on solid YESA, MEA, and CYA media under two temperature regimes designed to assess physiological plasticity: a condition supporting robust growth (25 °C for *P. pipericola* sp. nov. and 28 °C for *S. brasiliense* sp. nov.) and a condition imposing thermal stress, where growth was notably reduced (30 °C for *P. pipericola* sp. nov. and 40 °C for *S. brasiliense* sp. nov.). For each medium and condition tested, three agar plugs (5–7 mm^2^) were collected from each colony (edges and center), and homogenized in 3 mL of ethyl acetate/dichloromethane/methanol (3:2:1, *v*/*v*/*v*) containing 1% formic acid. Homogenates were sonicated for 50 min, filtered through 0.22 μm membranes, evaporated to dryness, and stored at −18 °C until further analysis.

The samples were received at the Compounds Management Laboratory (LGC/LNBio-CNPEM), where they were solubilized in DMSO (Sigma-Aldrich, Darmstadt, Germany, 99.7%), using the automated liquid handler Janus G3 (Revvity^®^, Waltham, MA, USA) to a final concentration of 10 mg/mL. Aliquots of 12 µL of each sample were plated in 384-well plates (Greiner Bio-One GmbH, Kremsmünster, Austria) for UPLC-MS/MS analysis.

A liquid chromatography (LC) method was utilized in an Acquity UPLC HClass system (Waters, MA, USA) coupled to a uHR-ESI-QqTOF Impact II (Bruker Daltonics, Billerica, MA, USA) tandem mass spectrometer, operating in the positive mode. The LC system was equipped with a C18 BEH Acquity 1.7 µm column 2.1 × 100 mm (Waters, MA, USA), operating at a flow rate of 0.5 mL/min and a column temperature of 40 °C. The elution gradient involved an initial mobile phase composed by 90% water, 5% acetonitrile (CH_3_CN) (Merck, Darmstadt, Germany), and 5% of 2% formic acid solution. At 10 min, a CH_3_CN gradient was performed (gradient curve 6 in the Acquity system) to increase the CH_3_CN concentration to 95%, while maintaining 5% of the 2% formic acid solution. At 12 min, the mobile phase was changed to 100% CH_3_CN (gradient curve 1 in the Acquity system). This was followed by 3 min of recalibration to the initial mobile phase, totaling 15 min elution time per analysis.

For mass spectrometry the electrospray source was set to the range of 30–2000 Da, a detection speed of 8 Hz 500 V end plate offset, a capillarity of 4500 V, nebulizer at 4.0 bar, and drying gas flow (nitrogen) at 10 L/min with a drying temperature of 200 °C. For MS/MS, the collision cell was 5.0 eV, with collision energy in the range of 20–70 V and an absolute fragmentation cutoff of 1000. Ions below 200 Da were excluded, and the “active exclusion” function was enabled. For internal calibration, 10 mM sodium formate solution was used. The raw data were converted to mzXML using the instrument’s software Bruker Compass Data Analysis v. 4.3.

Data obtained from LC-MS/MS (mzXML) were processed using NP^3^ MS Workflow software [37] with default parameters. The software utilizes the UNPD-ISDB [38] library as its built-in database for analysis. The consensus spectra generated from the NP^3^ MS Workflow (*.mgf files) were further searched against GNPS spectral libraries (https://gnps.ucsd.edu/ProteoSAFe/status.jsp?task=24ed5f80d0e74215800cdbed01080bb5 and https://gnps.ucsd.edu/ProteoSAFe/status.jsp?task=c8b8d3bf7ea74d24a19513ccdbe062b4, accessed on 16 June 2025) [39]. After processing in GNPS, the chemical annotations were grouped into the clean variable table, which resulted from NP^3^ MS Workflow softwmare processing. In the post-processing step, protonated ions (M+H^+^) were selected, along with a chemical curation procedure to determine the most appropriate chemical annotation (GNPS or UNPD), based on the parameters: MQScore, mzErrorPPM, and NumPeakShared. For GNPS, the following thresholds were applied: MQScore > 0.8, mzErrorPPM < 20, and NumPeakShared > 6; for UNPD-ISDB, the thresholds were: MQScore > 0.4, mzErrorPPM < 20, and NumPeakShared > 6.

## 3. Results and Discussion

**Figure 1:**
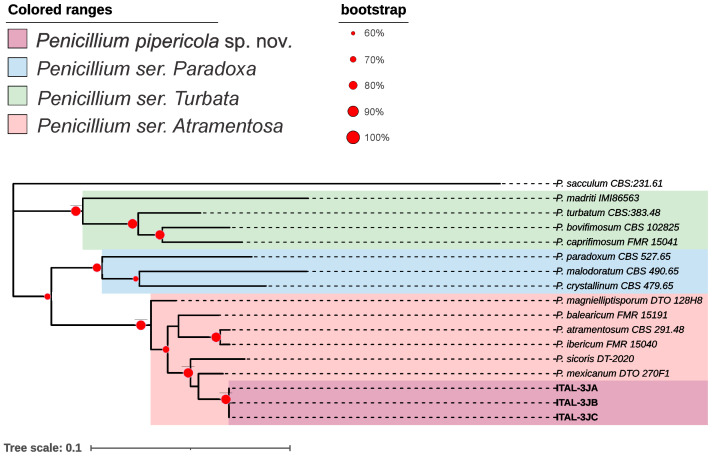
Maximum-likelihood tree (GTR+G+I) of *Penicillium* series *Paradoxa*, *Atramentosa* and *Turbata* based on combined dataset sequences (*CaM+BenA+RPB2+ITS*). Only bootstraps ≥ 60% are shown. *Penicillium sacculum* is the outgroup.

**Figure 2:**
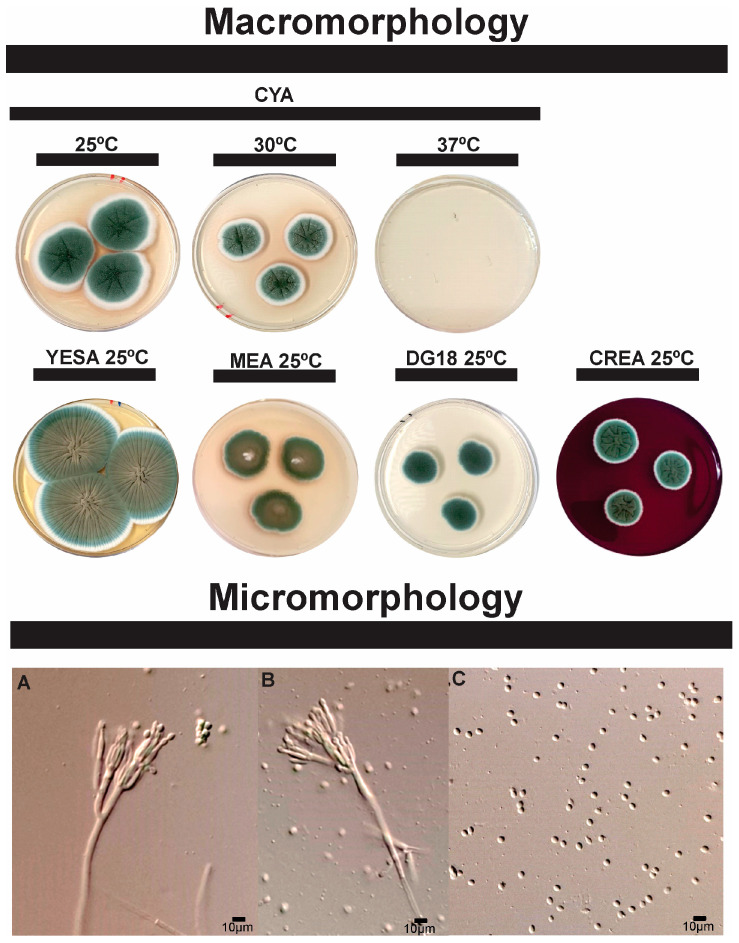
Morphological characters of *Penicillium pipericola* sp. nov. (ex-type ITAL-3JA). A colonies from left to right (top row) CYA (25 °C, 30 °C and 37 °C); (bottom row) YESA, MEA, DG18, and CREA (25 °C). Conidiophores (**A**,**B**), conidia (**C**).

**Figure 3:**
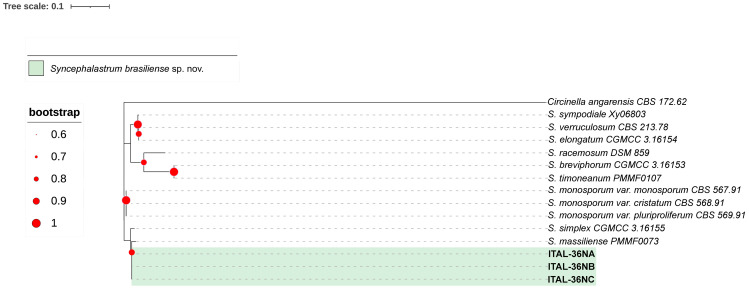
Maximum-likelihood tree (T93+G) of *Syncephalastrum* genus based on combined dataset sequences (*LSU*+*ITS*). Only bootstraps ≥ 60% are shown. *Circinella angarensis* is the outgroup.

**Figure 4:**
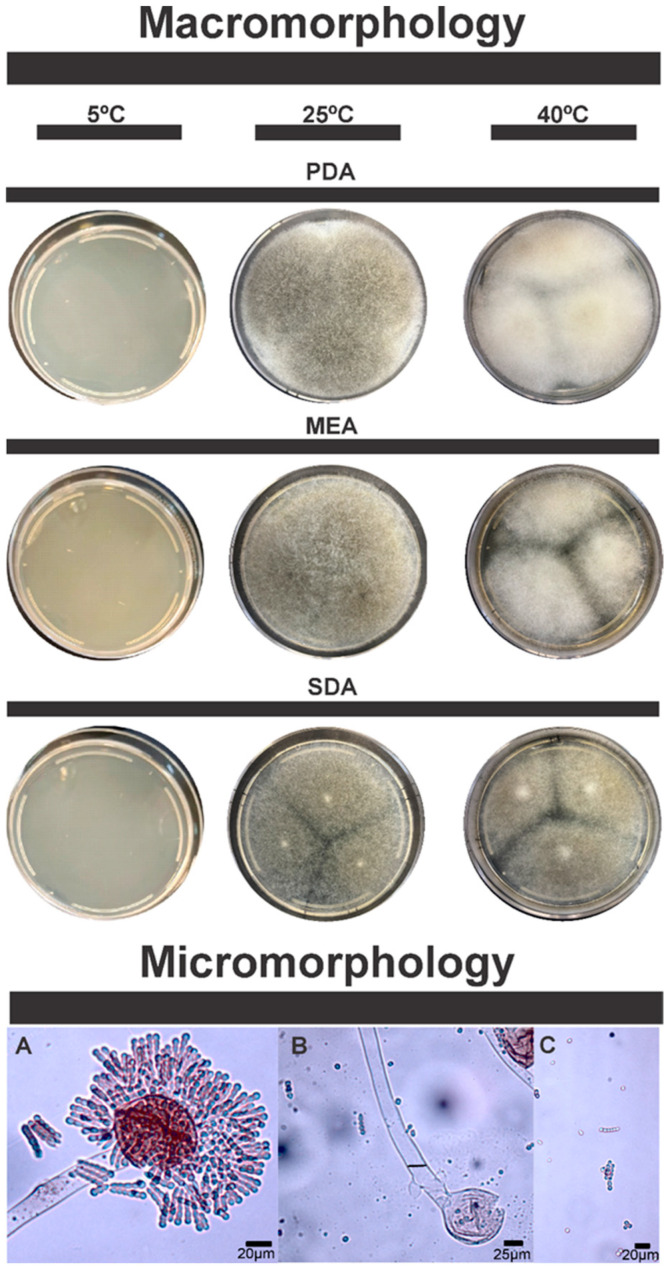
Morphological characters of *Syncephalastrum brasiliense* sp. nov. (ex-type ITAL-36NA). Columns from left to right represent different temperatures: 5 °C, 25 °C and 40 °C; Rows from top to bottom represent different media: PDA, MEA and SDCA+G. Sporangiophores and Merosporangium (**A**), Columella (**B**) and Sporangiospores (**C**).

The candidate species *Penicillium pipericola* sp. nov. (Figure 1 and Figure 2) and *Syncephalastrum brasiliense* sp. nov. (Figure 3 and Figure 4) have been identified as independent evolutionary lineages, each forming robust monophyletic groups as indicated by genealogical concordance. Individual phylogenetic trees for each analyzed locus are provided in Supplementary Material S2.

*Penicillium* is a large and diverse fungal genus with over 350 species distributed throughout the world in different environmental matrices, among which soils, plants, and food products stand out [40,41,42]. These fungi are important decomposers but are also capable of food spoilage and producing mycotoxins such as ochratoxin A and patulin [40,42], which are of concern for food safety. However, industrial applications for *Penicillium* are noted in cheese and sausage manufacturing and production of pharmaceutical important metabolites, including penicillin [40,41]. Recent genomic studies are providing some insight into the mechanisms of adaptation and horizontal gene transfer in *Penicillium* species [41]. Moreover, these fungi are promising in their biotechnological applications such as bioremediation, biofuel production, and food fermentation [43]. The versatility of *Penicillium* species makes them valuable objects for research into adaptation mechanisms and prospecting for metabolites of interest.

Some species of *Penicillium* have been shown to increase plant growth as well as resistance to pathogens. *Penicillium* culture filtrates increased seed germination and improved root and shoot growth in tomato and wheat [44,45]. Improvement in chlorophyll content, protein, and amino acid levels has been associated with some *Penicillium* strains in sesame plants [46]. These fungi have been shown to have protective effects against salt stress, as well to fungal pathogens such as *Fusarium* spp. [46]. *Penicillium* sp. strain GP15-1 has been shown to improve cucumber plants growth, and to protect them from damping off caused by *Rhizoctonia solani*, as well as anthracnose caused by *Colletotrichum orbiculare* [47].

The plant growth-promoting abilities of *Penicillium* spp. have been attributed to the production of beneficial metabolites, efficient root colonization, and induction of systemic resistance in host plants [44,47]. The isolation of *P. pipericola* sp. nov. from black pepper raises interest in investigating its ecological role in the black pepper production chain, its microbial interactions with other microorganisms, or with the plant host itself, and the production of mycotoxins by this species.

Analysis of the metabolomic profile of *P. pipericola* sp. nov. revealed a set of specialized metabolites whose production was modulated by variations in temperature and culture medium (Table 1). These compounds belong to different chemical classes, such as diketopiperazines, indole alkaloids, perylenequinones, and modified sterols, and reflect the biosynthetic plasticity of the studied strain when faced with different stimuli. Below we discuss some of the most relevant compounds that could be annotated in our analysis.

The altertoxin group of mycotoxins are perylene quinones, known to be produced by fungi of the genus *Alternaria* [48,49]. This group of mycotoxins can induce DNA strand breaks as they are highly mutagenic [48]. Altertoxins contaminate food and feed, especially after long storage periods, with altertoxins I, II, and III considered in the risk assessment of EFSA Panel on Contaminants in the Food Chain [50,51]. They are synthesized through the polyketide pathway, with the altertoxin I biosynthesis mechanism being proposed by Stinson [52]. Altertoxins can be produced in large quantities when *Alternaria* spp. are grown in modified Czapek-Dox medium with low glucose and ammonium sulfate [53]. Altertoxin II suppresses the immune response in the gastrointestinal tract and alters cell architecture in the colon [54], acting through the Nrf2-ARE pathway. Interestingly, the Nrf2-ARE pathway in mammalian cells is activated by altertoxin II (epoxyde), but not by its alcohol analog, altertoxin I [49]. Commercial food samples analyzed by Liu & Rychlik [53] contain alterotoxin I and II, but did not contain altertoxin III, although the latter is also an EFSA concern. However, some organic whole grains and paprika powder were contaminated with altertoxin I and another perylene derivative, alterperylenol. The highest concentrations of altertoxins were found in sorghum feed samples [53]. The finding that *Penicillium pipericola* sp. nov. occurs in Brazilian black pepper, and that this strain produces altertoxin III, highlights the importance to monitor altertoxins in this food ingredient.

Besides mycotoxins with potential concerns on food safety, we could also detect metabolites in *Penicillium pipericola* sp. nov. that are of interest in pharmaceutical development.

Myriberin A, which we found in *P. pipericola* sp. nov. (Table 1), is an alkaloid isolated from the plant *Myrioneuron faberi*, possessing an unprecedented heteropentacyclic skeleton [55]. It demonstrates inhibitory effects against the hepatitis C virus (HCV) life cycle in vitro [55,56]. To our knowledge, this is the first report of the production of this metabolite by a fungus.

Dehydrohistidyl-tryptophanyl-diketopiperazine (Table 1) is a fungal metabolite of the diketopiperazine (DKP) class, which in turn can be found in several microorganisms, particularly fungi and bacteria [57,58,59]. These compounds have varied chemical structures and biological activities, making them promising drug candidates [57,60]. The wide structural and bioactivity variation in indole DKPs, isolated mainly from *Aspergillus* and *Penicillium* species, has been the subject of extensive studies [57]. Some DKPs show potential in pest control, as observed in compounds from *Aspergillus ochraceus* [58]. Novel DKP derivatives, such as maremycins, have been found in *Streptomyces* sp., possessing unusual structural features such as spiro-indole groups. These compounds showed slight cytotoxicity against the mouse fibroblastoma cell line L-929, the human leukemia cell line K562, and the Hela human cervix carcinoma cell line [59]. Notably, 2,5-DKPs (such as those found in *P. pipericola* (Table 1)) have attracted considerable attention for their anticancer properties, and both natural and synthetic derivatives are being explored, particularly for use as anticarcinogenic agents [60].

Galangin (Table 1) is a flavonoid extracted from galangal and propolis. It has anti-inflammatory, antibacterial, and antioxidant activities [61]. Galangin inhibits cancer cell metastasis by blocking the PKC/ERK signaling pathways and reducing MMP-2/MMP-9 activity [62]. Furthermore, it stimulates the degradation of β-catenin, inhibiting the growth of colorectal and liver cancer cells [63]. In ovarian cancer, galangin can induce apoptosis through p53-dependent pathways, thus being selective for cancer cells, while sparing normal cells [64]. The anticarcinogenic effects are associated with the upregulation of pro-apoptotic proteins and the downregulation of the Akt/p70S6K pathways [64]. Although galangin shows promise as a potential therapeutic agent, further clinical and toxicity studies are needed to support its application [61].

Morphologically, *P. pipericola* sp. nov. exhibits distinct characteristics compared to its closest relative, *Penicillium mexicanum*. These differences include faster and broader growth patterns and a smoother colony appearance (Figure 2), while *P. mexicanum* has a grooved colony, which is not the case for *P. pipericola* sp. nov. The main comparable morphological characteristics between these two species are summarized in Table 2. The combined analysis of phenotypic and genotypic data justifies the classification of *P. pipericola* as a new species, placed in the subgenus *Penicillium*, section *Paradoxa*, series *Atramentosa*.

The genus *Syncephalastrum* is included in the group of zygomycetes, which play different ecological roles. First described by Thaxter in 1897 [65], *Syncephalastrum* species are generally known as environmental fungi and laboratory contaminants [66]. The genus is characterized by irregular, branched and aseptate hyphae with terminal vesicles surrounded by cylindrical merosporangia [66].

The genus *Syncephalastrum* belongs to the order Mucorales and has been increasingly reported in human infections, especially in immunocompromised individuals [66,67]. Two recently described species, *S. massiliense* and *S. timoneanum*, were isolated from clinical samples and presented unique phenotypic and genotypic characteristics [66]. *Syncephalastrum racemosum* is another member of this genus implicated in infections, known to cause highly invasive subcutaneous mucormycosis [67]. *S. brasiliense* sp. nov. is phylogenetically close to *S. massiliense*, which raises the need for further investigation into its potential as a pathogen in humans.

Similarly, *S. brasiliense* sp. nov. also shows clear morphological differences from its closest relatives, *Syncephalastrum massiliense* and *Syncephalastrum simplex*. *Syncephalastrum brasiliense* sp. nov. differs from related taxa by its ability to grow up to 45 °C. Colonies expand rapidly on MEA, PDA, and SDCA+G (40–70 mm/5 days), cottony to velvety, white to grayish, with pale reverse (Table 3).

Complementary metabolomic analyses showed that *S. brasiliense* sp. nov. exhibited considerable biosynthetic plasticity, producing diverse metabolites from different classes, many of which have biotechnological potential.

The compound Ac,Et ester-mandelic acid, detected in five of the six conditions (Table 1), is a derivative of mandelic acid, which in turn is a lipophilic α-hydroxy acid, which has antibacterial activity, in addition to the ability to inhibit sebocyte lipid synthesis, therefore being useful in the care of oily skin [68]. Some mandelic acid derivatives organize into chiral gels by self-assembly and can be used as templates for circularly polarized luminescent materials, applicable in the biomedical field [69]. Results of DPPH, FRAP, CUPRAC, and ABTS assays showed that mandelic acid and its derivatives have antioxidant activities [70]. Products containing mandelic acid have been found to improve parameters related to oiliness, shine, and signs of skin aging [68]. This profile makes mandelic acid suitable for cosmetic and pharmaceutical applications.

The metabolite 6-[3-[(3,4-dimethoxyphenyl)methyl]-4-methoxy-2-(methoxymethyl)butyl]-4-methoxy-1,3-benzodioxole NCGC00385811-01, a benzodioxol derivative, was detected in three of the six growth conditions tested (Table 1). Studies show that benzodioxole derivatives form stable metabolic complexes that can induce and modulate cytochrome P450 enzymes. Murray et al. [71] found a strong correlation between the enzyme-inducing ability of these compounds and aryl hydrocarbon hydroxylase activity (r = 0.980). Later, Kumagai et al. [72] confirmed that these complexes form and have characteristic absorption properties at 455 nm. They also found that these complexes inhibit the catalytic cycle of enzymes, which helped to better understand how these mechanisms work. The metabolism of benzodioxoles includes oxidation to catechols and the generation of carbon monoxide derived from the methylene carbon atom [73]. Some benzodioxole derivatives exhibit anticancer potential, particularly against hepatocellular carcinoma cells, as they reduce alpha-fetoprotein secretion and induce cell cycle arrest [74]. Furthermore, some benzodioxole-derived compounds exhibit antioxidant properties [74]. The unique structure of benzodioxoles leads to pharmacokinetic interactions and potential toxicity through a quinone-reactive oxygen mechanism [72].

The flavone 7,8-dihydroxyflavone (DHF) (Table 1) is a natural flavonoid with diverse therapeutic potential. As a high-affinity TrkB agonist, DHF exhibits neuroprotective effects against several central nervous system diseases, including Alzheimer’s disease and Parkinson’s disease [75]. In the ICV-STZ mouse model of sporadic Alzheimer’s disease, DHF improved cognitive function by alleviating oxidative stress, mitochondrial dysfunction, and insulin resistance [76]. In addition to its neuroprotective effects, DHF also exhibits anti-aging effects in human dermal fibroblasts by inducing collagen synthesis, inhibiting MMP-1 expression, and reducing intracellular reactive oxygen species. It also upregulates antioxidant enzymes and modulates the MAPK/Akt signaling pathway, which is involved in skin aging [77]. Overall, these studies highlight the potential of DHF as a nutraceutical for the treatment of neurological diseases and as an anti-aging skin agent.

The analysis of phenotypic and genotypic data confirms that *S. brasiliense* sp. nov. is a new species within the order Mucorales, family Syncephalastraceae, and genus *Syncephalastrum*.

Therefore, the detailed descriptions of these new species, *P. pipericola* sp. nov. and *S. brasiliense* sp. nov., are based on a robust phylogenetic analysis and the observation of unique morphological and physiological characteristics that definitively differentiate them from related taxa.

## 4. Taxonomy

***Penicillium pipericola* sp. nov.** Rosa, V.S.; Taniwaki, M.H.; Silva, J.J.

**Mycobank**: MB861249

**Etymology**: The specific epithet refers to the substrate from which it was isolated, black pepper, *Piper nigrum*.

In *Penicillium* subgen. *Penicillium* sect. *Paradoxa* ser. *Atramentosa.*

**Typification**: BRAZIL. São Paulo State, Campinas City, 22°54′06.7″ S 47°03′37.2″ W, in black pepper, 10 April 2024, isolated by Rosa, V.S. Holotype: CIMFI 3JA, preserved as a metabolically inactive culture. Ex-type culture: ITAL-3JA.

**DNA barcodes**: *BenA* (PV022467), *CaM* (PV022465), *RPB2* (PV022466), ITS (PV007905).

**Colony diam.**: 7 days, 25 °C: CYA 38.3–43.0 mm, MEA 30.0–33.0 mm, YESA 47.0–51.0 mm; DG18 19.0-26.0 mm; CREA 19.6-21.0 mm; CYA 30 °C 28.0–33.0 mm.

**Diagnosis**: Morphologically, *P. pipericola* sp. nov. can be differentiated from *P. mexicanum* by its significantly faster growth on several media: CYA, YESA and MEA. Its colony morphology is also notably distinct; the colony surface of *P. pipericola* is velvetier with a radial structure, while *P. mexicanum* tends to have deep and highly sulcate colonies (see description of *P. mexicanum* Visagie et al. [78]. Phylogenetically, *P. pipericola* sp. nov. is easily distinguished from *P. mexicanum* and other species in ser. *Atramentosa*. The main distinguishing features compared to the type strain of *P. mexicanum* (CBS H-21805) are: 9 single-nucleotide polymorphisms (SNPs) in the *BenA* locus, 16 SNPs in *RPB2*, 24 SNPs in *CaM*, and 2 SNPs in ITS. When the *CaM*+*ITS*+*RPB2*+*BenA* genealogies are combined, *P. pipericola* sp. nov. forms a well-supported independent evolutionary lineage (IEL) with a bootstrap value of 90% (Figure 1).

**Colony characters**: On CYA 25 °C, green colonies, with white edges, abundant sporulation, velvety surface with radial juices, exudates present; soluble pigments and sclerotia absent. In MEA 25 °C, 7 days: Small colonies with a white center and a gradient from brown to green towards the edges. Irregular and thin edges, smooth surface. Pigments, exudates and sclerotia absent. In YESA 25 °C, 7 days: Large colonies with brownish center with gradient of green tones towards the edges; velvety appearance; abundant and ordered radial grooves with wrinkled center. Soluble pigments, exudates and sclerotia absent. In DG18 25 °C, 7 days, Small, flat colonies with velvety appearance, white edges; uniform green color throughout the colony. Soluble pigments, exudates and sclerotia absent. In CREA 25 °C, 7 days, small colonies, green in color, pronounced center with cerebrospinal appearance, smooth rounded edges, without acid production.

**Micromorphology**: Conidiophores triverticillate; Stipes smooth-walled, 31.2 × 3.1 μm; Metulae, 10.6 × 3.0 μm (7.7 − 16.5 × 2.9 − 3.5); Phialides ampulliform, 10.0 × 2.4 μm (9.9 − 12.5 × 2.2 − 3.5); Conidia smooth and ellipsoidal 3.3 × 2.9 μm (2.9 − 4.7 × 2.7 − 3.0).

***Syncephalastrum brasiliense* sp. nov.** Rosa, V.S.; Iamanaka, B.T.; Silva, J.J.

**Mycobank**: MB861250.

**Etymology**: Named after its place of isolation, Brazil.

In gen. *Syncephalastrum* fam. Syncephalastraceae ordo. Mucorales cl. Mucoromycetes subdiv. Mucoromycotina div. Mucoromycota subregn. Mucoromyceta.

**Typification**: BRAZIL. São Paulo State, Campinas City, 22°54′06.7″ S 47°03′37.2″ W, in black pepper, 10 April 2024, isolated by Rosa, V.S. Holotype: CIMFI 36NA, preserved as a metabolically inactive culture. Ex-type culture: ITAL-36NA.

**DNA barcodes**: ITS (PV007760); LSU (PV015155).

**Colony diam.**: 7 days, 25 °C: MEA 40.0–68.0 mm, PDA 44.0–67.0 mm, SDCA+G 41.0–70.0 mm.

**Diagnosis**: Morphologically, *S. brasiliense* can be distinguished from *S. massilense* primarily by its robust growth at 40 °C (with growth observed at 45 °C), sporangiospore size, and colony coloration (see the description of *S. massilense* by Kabtani et al. [66]). It differs from *S. simplex* in colony texture and coloration, as well as vesicle shape (see the description of *S. simplex* by Zhao et al. [79]). Phylogenetically, the main distinguishing features compared to the type strain of *S. massilense* (CBS H-21805) are: 6 single-nucleotide polymorphisms (SNPs) and 1 INDEL (insertion/deletion) in the ITS locus, as well as 5 INDELs in the LSU locus. When the ITS+LSU genealogies are combined, *S. brasiliense* sp. nov. forms a well-supported independent evolutionary lineage (IEL) with a bootstrap value of 82% (Figure 3).

**Colony characters**: On MEA 25 °C, rapid and expansive growth, covering the entire plate in 5 days; homogeneous white to cream coloration, some strains may develop grayish tones from the fifth day onwards; cottony and aerial texture, typical of Mucorales fungi, with long and diffuse hyphae, indistinct margin, diffuse growth without clear delimitation. reverse of the plate pale, colonies between 40–68 mm. Grows well at 40 °C, but there is no growth at 5 °C. In PDA 25 °C, rapid and expansive growth, covering the entire plate in 5 days, white to light gray coloration, with a slightly darker center, suggesting the beginning of sporulation. Cottony and densely aerial texture, with diffuse and filamentous mycelium; diffuse and irregular margin, with homogeneous radial growth; reverse of the plate pale to yellowish brown. Colonies between 44–67 mm. Grows well at 40 °C, but no growth at 5 °C; exudates are produced. On SDCA+G 25 °C, rapidly growing colony covering nearly the entire surface of the plate; predominantly white to grayish in color, with a slight darkening in the center. Flaky, densely filamentous texture, with a velvety or cottony appearance. Well-defined margins, which may be slightly diffuse due to the rapid expansion of the mycelium. Reverse of the plate pale to light, colonies 41–70 mm, with aerial hyphae. Grows well at 40 °C, but no growth at 5 °C.

**Micromorphology**: Sporangiophores erect, branched or simple, sporangia of variable shape, mainly subglobose, born from aerial, coenocytic, smooth and hyaline hyphae. Merosporangium present with globose to subglobose vesicle covered radially by cylindrical merosporangia lined up on the surface of the vesicle. Sporangiospores with smooth surface, elliptical, globose and subglobose, individual or in chains of up to 7 sporangiospores. Zygospores and chlamydospores not observed. Sporangia, 41–70 μm; hyphae 11–24 μm wide; merosporangia 6–11 μm; sporangiospores, 4–7 μm.

## 5. Conclusions

This study describes two new species, *Penicillium pipericola* sp. nov. and *Syncephalastrum brasiliense* sp. nov., isolated from Brazilian black pepper. The integration of morphological, phylogenetic, and metabolomic data supports their proposal as new species. The presence of these fungi in black pepper highlights the importance of microbiological monitoring, both for food safety purposes and for expanding our understanding of ecological interactions and biotechnological applications of these fungi. These results prompt further investigations to clarify the impact of these species on product quality and their role in the microbial dynamics of the agroecosystem. Furthermore, the metabolomic profile provides a promising perspective for future investigations into the biotechnological potential of these two new species.

## Figures and Tables

**Table 1:** Metabolomic Characterization of *Penicillium pipericola* sp. nov. and *Syncephalastrum brasiliense* sp. nov. under Variable Growth Conditions. Annotated metabolites were considered according to the spectra annotation parameters and thresholds described in the *Material and Methods* section.

**Metabolites Produced by *Penicillium pipericola* sp. nov.**	**Growing Conditions**					
	**25 °C MEA**	**25 °C CYA**	**25 °C YESA**	**30 °C YESA**	**30 °C MEA**	**30** **°C CYA**
(3E)-2,6,12-Trihydroxy-3-(1H-imidazol-5-ylmethylene)-7a-(2-methyl-3-buten-2-yl)-7a,12-dihydro-3H,5H-imidazo [1′,2′:1,2]pyrido [2,3-b]indol-5-one	+	-	+	+	+	+
(+)-6-hydroxymellein diacetate	+	-	+	+	+	-
(14alpha,22E)-14-hydroxyergosta-7,22-diene-3,6-dione	+	-	-	+	-	-
(20S,22E)-24-methylcholesta-1,4,22-triene-18,20-diol-3-one	-	-	+	-	-	-
(3beta,5beta,6beta,7alpha,11alpha)-5,6-Epoxyergosta-8,24(28)-diene-3,7,11-triol	-	+	+	-	+	+
(3beta,8(,9(,15(,24()-8,9-Epoxyergosta-5,22-diene-3,15-diol	+	-	+	+	+	+
(3R, 7aS)-Cyclo(histidylproline	-	-	-	+	-	-
(3Z)-6-Hydroxy-3-(1H-imidazol-5-ylmethylene)-12-methoxy-7a-(2-methyl-3-buten-2-yl)-7a,12-dihydro-1H,5H-imidazo[1′,2′:1,2]pyrido[2,3-b]indole-2,5(3H)-dione	+	+	+	+	+	+
(6-methyl-ergolin-8beta-yl)-methanol	-	-	+	-	-	-
(9S,10R,11R, 12Z,15Z)-9,10,11-trihydroxyoctadeca-12,15-dienoic acid	+	-	+	+	-	-
(R)-2′-hydroxy-justirumalin	+	-	-	-	-	-
1-palmitoyl-sn-glycero-3-phosphoethanolamine	+	-	+	+	-	+
1,8,15,22-Tetraaza-cyclooctacosan-2,9,16,23-tetraon	+	+	+	+	+	+
12,13-epoxy-alpha-santalene	-	-	+	+	-	-
14,15-bisnor-3,11E-kolavadien-13-one	-	-	+	+	+	-
23-nor-spiculoic acid B	-	-	+	-	-	-
24-exomethylenecalicoferol E	-	-	+	+	+	-
24-methylenecholesta-5,7,9(11)-trien-3beta-ol	+	-	+	+	+	+
2alpha-methoxyhomolycorine	-	-	+	-	+	-
3-(1H-Imidazol-4-ylmethyl)-10b-(2-methyl-3-buten-2-yl)-6,10b,11,11a-tetrahydro-2H-pyrazino[1′,2′:1,5]pyrrolo[2,3-b]indole-1,4(3H,5aH)-dione	-	-	+	+	+	-
3-(1H-Imidazol-4-ylmethyl)-6-(1H-indol-3-ylmethyl)-2,5-piperazinedione	-	-	+	+	-	-
3-(indol-3-yl)quinoline	-	-	+	+	-	-
3,5,7,8-Tetrahydroxyflavone	-	-	-	-	+	-
5-hydroxy-3-(4-hydroxyphenyl)-7-[(2S,3R,4S,5S,6R)-3,4,5-trihydroxy-6-(hydroxymethyl)oxan-2-yl]oxychromen-4-one	+	-	-	-	+	-
5alpha,6alpha-epoxy-(22E)-ergosta-8,14,22-triene-3beta,7alpha-diol	+	+	-	-	+	+
5R)-3-({(1S,2R,4aS,6R,8aR)-1,6-Dimethyl-2-[(1E)-1-propen-1-yl]-1,2,4a,5,6,7,8,8a-octahydro-1-naphthalenyl}carbonyl)-4-hydroxy-5-(hydroxymethyl)-1-methyl-1,5-dihydro-2H-pyrrol-2-one	+	+	+	+	+	+
7-hydroxy-6-methoxy-8-acetyl-2H-<1>-benzopyran-2-one	-	-	+	+	-	-
7,8-Dihydroxyflavone	+	-	-	-	+	-
8-O-beta-D-(6′-O-acetyl)glucopyranosyl-chrysophanol	+	-	-	-	-	-
Ac-8-Hydroxy-3,4-dimethyl-1H-2-benzopyran-1-one	+	-	+	+	-	+
Altertoxin III	+	-	+	+	+	+
Androsta-11,15-diene-14-carboxylic acid, 3-(acetyloxy)-6,19-epoxy-15-hydroxy-4,4,8,12,16-pentamethyl-17,19-dioxo-, methyl ester, (3alpha,6alpha,9xi,14beta)-	+	+	+	+	+	+
Benzyl butyl phthalate	+	-	-	+	-	-
Chabrolosteroid G	-	-	-	+	-	-
Chanoclavin I-saeure	-	-	+	+	-	-
Citreoanthrasteroid B	+	-	+	+	+	+
Clavicipitic acid	+	-	+	+	-	-
D-Phe-L-Val-D-Val-L-Tyr	-	-	+	+	+	-
Dalmanol A	+	-	+	+	+	+
Dehydrohistidyl-tryptophanyl-diketopiperazine	-	-	+	+	+	+
Desmethylaltenusin	-	-	+	+	-	-
Dihydrocochloxanthin	-	-	+	+	-	-
Dihydrogambirtannine	+	-	+	-	-	-
Galangin	+	-	-	-	+	-
Isoepijuvabiol	+	-	+	+	-	-
Juglomycin H	-	-	+	+	-	-
Lysergine	-	-	+	-	-	-
Mammea A/BA cyclo F	-	-	+	+	-	-
Melodinine E	-	-	+	-	-	-
Methyl (3beta,5beta,8alpha,9beta,10alpha,13alpha)-3-acetoxy-4,4,8,12,16-pentamethyl-15,17,19-trioxoandrost-11-ene-14-carboxylate	-	-	+	+	-	-
Methyl 6,10-dimethylundecanoate	-	-	-	+	-	-
Myriberine A	+	-	+	+	+	+
N-(2-indol-3-yl-ethyl)-N-methyl-formamide	+	-	+	+	-	-
N-prenyl-cyclo-L-tryptophyl-L-proline	-	-	+	+	-	-
4-[5-[[4-[5-[acetyl(hydroxy)amino]pentylamino]-4-oxobutanoyl]-hydroxyamino]pentylamino]-4-oxobutanoic acid	+	-	+	+	+	+
Panicein E	-	-	+	-	-	-
Persicaxanthal	+	-	+	+	-	-
Quebrachamin	+	-	-	-	-	-
Retusin	+	-	-	-	+	-
Roquefortine M	+	-	+	+	+	+
Schizozygine	-	-	+	-	-	-
Serratin 7-beta-glucoside	+	-	-	-	-	-
Solajiangxin E	+	-	-	-	-	-
Tropolactone A	-	-	+	-	-	-
**Metabolites produced by *Syncephalastrum brasiliense* sp. nov.**						
	**28 °C YESA**	**28 °C MEA**	**28 °C CYA**	**40 °C YESA**	**40 °C MEA**	**40 °C CYA**
(1′R,2′S)-candenatenin D	-	-	+	-	-	-
(14alpha,22E)-14-hydroxyergosta-7,22-diene-3,6-dione	-	-	-	+	-	-
(14RS)-(10E,12E)-14-hydroxy-9-oxo-10,12-octadecadienoic acid	-	-	+	-	-	-
(2E,6E)-3-formyl-7-methyl-9-(2,6,6-trimethylcyclohex-2-enyl)nona-2,6-dienyl acetate	-	+	-	-	-	-
(3beta,8(,9(,15(,24()-8,9-Epoxyergosta-5,22-diene-3,15-diol	-	+	+	+	+	+
(5beta,7beta,20xi,22E)-11,20-Dihydroxy-23-methylcholesta-1,22-dien-7-one	-	-	-	+	-	-
(5E,9E,13R)-13,14-dihydroxy-6,10,14-trimethylpentadeca-5,9-dien-2-one	-	-	+	-	-	-
(7E)-9-ketooctadec-7-enoic acid	-	+	+	-	-	-
(R)-11-Cycloheptyl-2-hydroxyundecanoic acid	-	+	-	-	-	-
1-methyl-2-tetradecyl-4(1H)-quinolone	-	-	+	-	-	-
1-palmitoyl-sn-glycero-3-phosphoethanolamine	-	+	+	+	+	+
12-hydroxy-9Z,13E-octadecadienoic acid	-	+	+	-	-	-
13-hydroxystearic acid	-	-	-	-	-	+
18-Hydroxy-9,11,13-octadecatrienoic acid	-	+	+	-	-	+
2-amino-1,3,4-octadecanetriol	-	-	+	+	+	+
2-Amino-4-heptadecene-1,3-diol	-	-	-	+	-	-
2,3-Dihydroxy-2-(1-hydroxytridecyl)-4-methoxycyclopentanone	-	-	+	-	-	-
24-Hydroxy-11-deoxoglycyrrhetic acid	-	-	-	-	+	-
24-methylenecholesta-5,7,9(11)-trien-3beta-ol	-	+	+	+	-	-
3-3,25-Dihydroxyergosta-5,24(28)-dien-7-one	-	-	-	-	+	-
3-hydroxyheteroendrin	+	+	-	-	+	-
4-[5-[[4-[5-[acetyl(hydroxy)amino]pentylamino]-4-oxobutanoyl]-hydroxyamino]pentylamino]-4-oxobutanoic acid	-	+	+	+	-	+
4-Hydroxy-beta-snyderol	-	-	+	-	-	-
4′,7,8-Trihydroxyflavone	-	+	-	+	-	-
5-hydroxy-3-(4-hydroxyphenyl)-7-[(2S,3R,4S,5S,6R)-3,4,5-trihydroxy-6-(hydroxymethyl)oxan-2-yl]oxychromen-4-one	-	+	-	+	-	-
5-methoxy-2-oxoindolin-3-acetic acid methyl ester	-	+	-	-	-	-
5,6,7-Trihydroxyisoflavone	-	+	-	-	-	-
6-[3-[(3,4-dimethoxyphenyl)methyl]-4-methoxy-2-(methoxymethyl)butyl]-4-methoxy-1,3-benzodioxole	-	+	+	+	-	-
6-Geranyl-4-hydroxy-3-(2-hydroxypropyl)-2-pyrone	-	-	+	-	-	-
6′-0-acetylgenistin	-	+	-	+	-	-
7,8-Dihydroxyflavone	-	+	+	+	-	-
9,10-Epoxy-18-hydroxy-12-octadecenoic	-	-	+	-	-	-
Ac,Et ester-Mandelic acid	-	+	+	+	+	+
Benzyl butyl phthalate	-	+	+	+	-	-
Chokol F	-	-	+	-	-	-
Chrysophanein	-	+	-	+	-	-
Citreoanthrasteroid B	-	+	-	+	-	-
Cyclo-Tetrakis-epsilon-aminocaproyl	-	+	+	+	+	+
Cynarinin B	-	+	-	-	-	-
Dehydrobrevicollin	-	-	-	-	-	+
Emodin-8-O-beta-D-((6)-O-acetyl)glucopyranoside	-	+	-	+	-	-
Fructoselysine	-	-	-	+	-	-
Glyceryl monolinolenate	-	-	+	-	-	-
Lambertsaeure	-	+	-	-	-	-
Linoleamide	-	-	+	+	+	+
Retusin	-	+	-	+	-	-
Solajiangxin E	-	-	+	-	-	-
Tedanin	-	-	+	-	-	-
Trans,trans-1,7-diphenylhepta-4,6-dien-3-one	+	-	-	-	-	-
Tuberatolide A	-	-	+	-	-	-
Tumonoic acid D	-	-	-	+	-	-
Wedelia-secco-kaurenolide	-	+	+	-	-	-

**Table 2:** Main morphological characteristics comparable between *Penicillium pipericola* sp. nov. and *Penicillium mexicanum*.

Media (25 °C, 7 d)	*Penicillium pipericola* sp. nov.	*Penicillium mexicanum* (See The Original Description)
**CYA**	Green colonies with white edges, abundant sporulation, velvety surface with radial grooves, exudates present; soluble pigments and sclerotia absent.	Colonies moderately deep, sulcate; margins low, narrow, in some isolates irregular; mycelia white; texture velutinous; sporulation moderately dense, conidia en masse greyish green; soluble pigments absent; exudates abundant, clear to purplish.
**MEA**	Small colonies with white center and gradient from brown to green towards the edges; irregular and thin edges, smooth surface; soluble pigments, exudates and sclerotia absent.	Colonies low, radially sulcate, raised at centre; margins low, narrow, irregular; mycelia white; texture velutinous; sporulation dense, conidia en masse greyish green; soluble pigments absent; exudates absent.
**YESA**	Large colonies with brownish center with gradient of green tones towards the edges; velvety appearance; abundant and ordered radial grooves with wrinkled center; soluble pigments, exudates and sclerotia absent.	Colonies moderately deep, randomly sulcate, raised at centre; margins low, narrow, irregular; mycelia white; texture velutinous; sporulation moderately dense, conidia en masse dull green; soluble pigments absent; exudates absent.
**DG18**	Small, flat colonies with velvety appearance, white edges; uniform green color throughout the colony; soluble pigments, exudates and sclerotia absent.	Colonies low, very lightly radially sulcate; margins low, narrow, entire; mycelia white; texture velutinous; sporulation moderately dense, conidia en masse greyish green; soluble pigments absent; exudates absent.
**CREA**	Small colonies, green in color, pronounced center with cerebrospinal appearance, smooth rounded edges, without acid production.	Acid not produced.

**Table 3:** Main morphological characteristics of *Syncephalastrum brasiliense* sp. nov. and the closest species.

Characteristic and Media	*S. brasiliense* sp. nov.	*S. massiliense* (See The Original Description)	*S. simplex* (See The Original Description)
**Growth at high temperatures**	Grows well at 40 °C, growth observed up to 45 °C	Optimal growth at 25 °C; inhibited ≤4 °C and ≥40 °C	No growth reported ≥40 °C
**Growth at low temperatures**	No growth at 5 °C	Inhibited ≤4 °C	Not reported
**Sporangiospores**	With smooth surface, elliptical, globose and subglobose, individual or in chains (4–7 μm)	Smooth-walled and spherical to ovoid (3–6 μm)	With striation, variable shape, mainly globose, subglobose, ovoid or ellipsoid (3.0–7.5 μm)
**MEA**	Fast-growing (40–68 mm/5 days), white-cream to grayish, cottony colonies; pale reverse. Grows at 40 °C, not at 5 °C.	Not described	Not described
**PDA**	Rapid growth (44–67 mm/5 days), white to light gray with darker center; cottony, densely aerial mycelium; pale to yellowish-brown reverse; exudates present.	Colonies with fluffy cottony aspect; optimal growth at 25 °C, inhibited ≤4 °C or ≥40 °C; white at 48 h, darker at 72 h, high sporulation around day 5	90 mm; lobed, floccose, granulate; initially white, soon becoming Clove Brown; reverse irregular; abundant growth
**SDCA+G**	Rapid growth (41–70 mm), white to grayish with darker center; flaky to cottony, velvety texture; well-defined to slightly diffuse margins; pale reverse; grows at 40 °C, not at 5 °C.	Cottony, fluffy colonies; rapid growth at 25 °C, inhibited ≤4 °C or ≥40 °C; white (48 h), darker at 72 h, abundant sporulation after 5 days	Not described

## Data Availability

The sequences newly generated in this study have been submitted to the GenBank database. Additional data are available upon request from the corresponding authors.

## Supplementary Materials

The following supporting information can be downloaded at: https://www.mdpi.com/article/10.3390/microorganisms13122691/s1, Supplementary Material S1: Table showing the GenBank accession numbers used in the phylogenetic analyses conducted in this study. Table (A) Accessions included in the phylogenetic analysis presented in Figure 1. Table (B) Accessions included in the phylogenetic analysis presented in Figure 3; Supplementary Material S2: Phylogenetic trees of maximum likelihood for each locus individually. Figures S2A–S2D (*Penicillium pipericola* sp. nov.); Figures S2E–S2G (*Syncephalastrum brasiliense* sp. nov.).
