# Supplementary material for: Newly Discovered Fungal Species from Black Pepper Marketed in Brazil: Penicillium pipericola sp. nov. and Syncephalastrum brasiliense sp. nov
Source: Microorganisms. 2025 Nov 25;13(12):2691. doi: 10.3390/microorganisms13122691 (PMC12735095; doi:10.3390/microorganisms13122691)
Supplement: Supplementary file 1 [file microorganisms-13-02691-s001.zip › Supplementary Material S1-ROSA MO 2025.pdf]

Supplementary Material S1. Table showing the GenBank accession numbers used in the phylogenetic analyses conducted in this study. Table (A) Accessions included in the phylogenetic analysis presented in Figure 1. Table (B) Accessions included in the phylogenetic analysis presented in Figure 3.

Table (A)

	ITS	*CaM*	*BenA*	*RPBII*
Penicillium balearicum	LT899762.1	LT899758.1	LT898227.1	LT899760.1
Penicillium mexicanum	KJ775685.1	KJ775412.1	KJ775178.1	MN969127.1
Penicillium magnielliptisporum	KJ775686.1	KJ775413.1	KJ775179.1	MN969124.1
Penicillium paradoxum	EF669707.1	EF669692.1	EF669683.1	EF669670.1
Penicillium crystallinum	AF033486.1	FJ530973.1	EF669682.1	EF669669.1
Penicillium malodoratum	AF033485.1	FJ530972.1	EF669681.1	EF669672.1
Penicillium ibericum	LT899782.1	LT899766.1	LT898285.1	LT899800.1
Penicillium caprifimosum	LT899781.1	LT899765.1	LT898238.1	LT899799.1
Penicillium atramentosum	AF033483.1	KU896821.1	AY674402.1	JN406584.1
Penicillium turbatum	AF034454.1	KU896853.1	KJ834499.1	JN406556.1
Penicillium madriti	AF033482.1	EU644076.1	KJ834470.1	JN406561.1
Penicillium bovifimosum	AF263347.1	FJ530989.1	KJ834436.1	JN406649.1
Penicillium sicoris	LR884497.1	LR884496.1	LR884494.1	LR884495.1
Penicillium pipericola sp. nov.	PV007905.1	PV022465.1	PV022467.1	PV022466.1
Penicillium sacculum	KC411707.1	KU896849.1	KJ834488.1	JN121462.1

Table (B)

	ITS	*LSU*
Syncephalastrum massiliense	OL699905.1	OM417069.1
Syncephalastrum simplex	OL678220.1	NG_244100.1
Syncephalastrum monosporum var. pluriproliferum	OL699910.1	OM417074.1
Syncephalastrum monosporum var. cristatum	OL699909.1	OM417073.1
Syncephalastrum monosporum var. monosporum	OL699908.1	OM417072.1
Syncephalastrum timoneanum	OL699906.1	OM417070.1
Syncephalastrum breviphorum	OL678217.1	NG_244099.1
Syncephalastrum racemosum	OL699907.1	OM417071.1
Syncephalastrum elongatum	OL678219.1	PQ399930.1
Syncephalastrum verruculosum	HM999978.1	MH872886.1
Syncephalastrum sympodiale	OL678222.1	PQ399932.1
Syncephalastrum brasiliense sp. nov.	PV007760.1	PV015155.1
Circinella angarensis	JN205848.1	MH869719.1
