# Supplementary material for: Newly Discovered Fungal Species from Black Pepper Marketed in Brazil: Penicillium pipericola sp. nov. and Syncephalastrum brasiliense sp. nov
Source: Microorganisms. 2025 Nov 25;13(12):2691. doi: 10.3390/microorganisms13122691 (PMC12735095; doi:10.3390/microorganisms13122691)
Supplement: Supplementary file 1 [file microorganisms-13-02691-s001.zip › Supplementary Material S2-ROSA MO 2025.pdf]

**Supplementary Material S2**. Phylogenetic trees of maximum likelihood for each locus individually. Figures 2A-2D (*Penicillium pipericola* sp. nov.); Figures 2E-2G (*Syncephalastrum brasiliense* sp. nov.).

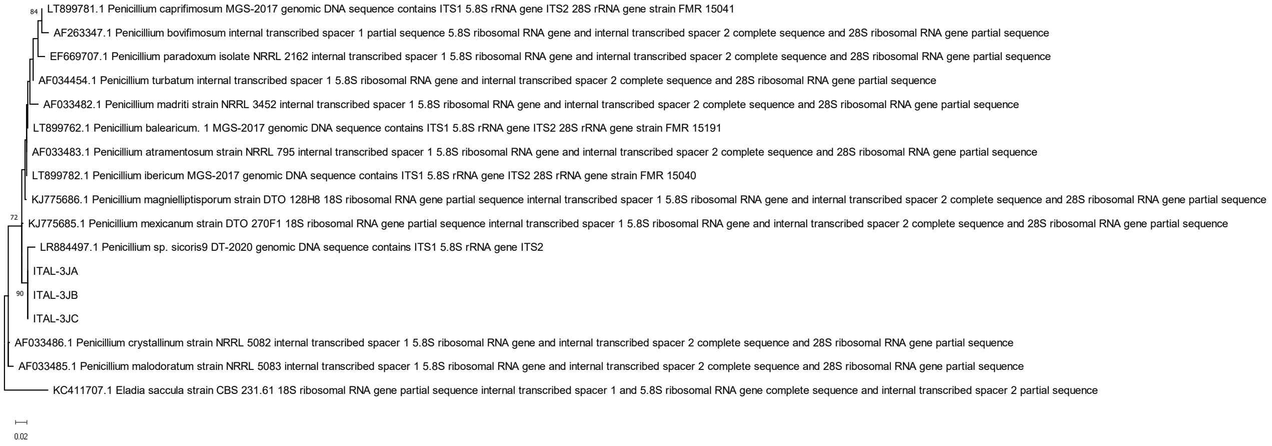


**Figure S2A.** Maximum-likelihood tree (GTR+G+I) of *Penicillium* series: *Paradoxa, Atramentosa* and *Turbata* based on *ITS* data sequences. Only bootstraps ≥ 60% are shown. *Penicillium sacculum* is the outgroup.

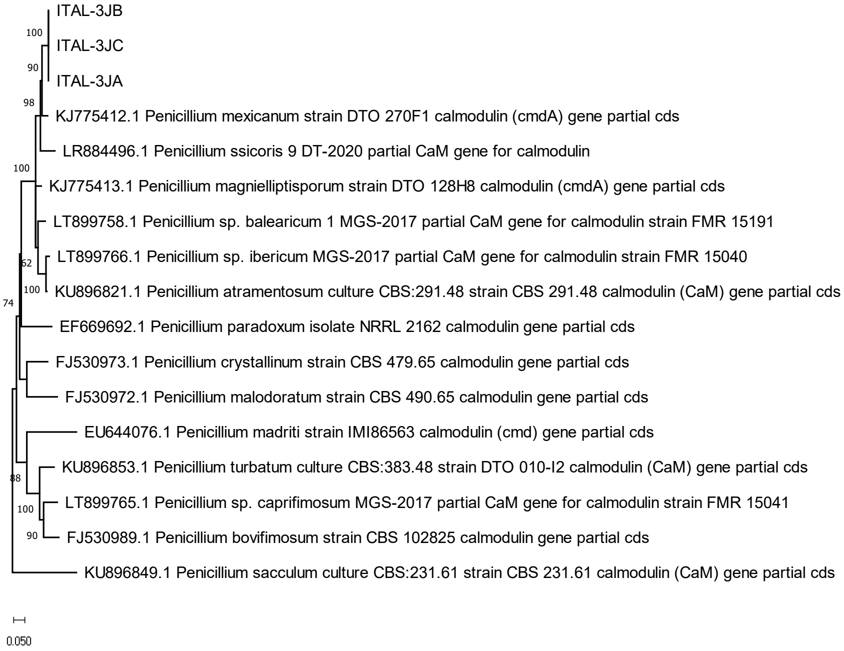


**Figure S2B.** Maximum-likelihood tree (GTR+G+I) of *Penicillium* series: *Paradoxa, Atramentosa* and *Turbata* based on *CaM* data sequences. Only bootstraps ≥ 60% are shown. *Penicillium sacculum* is the outgroup.

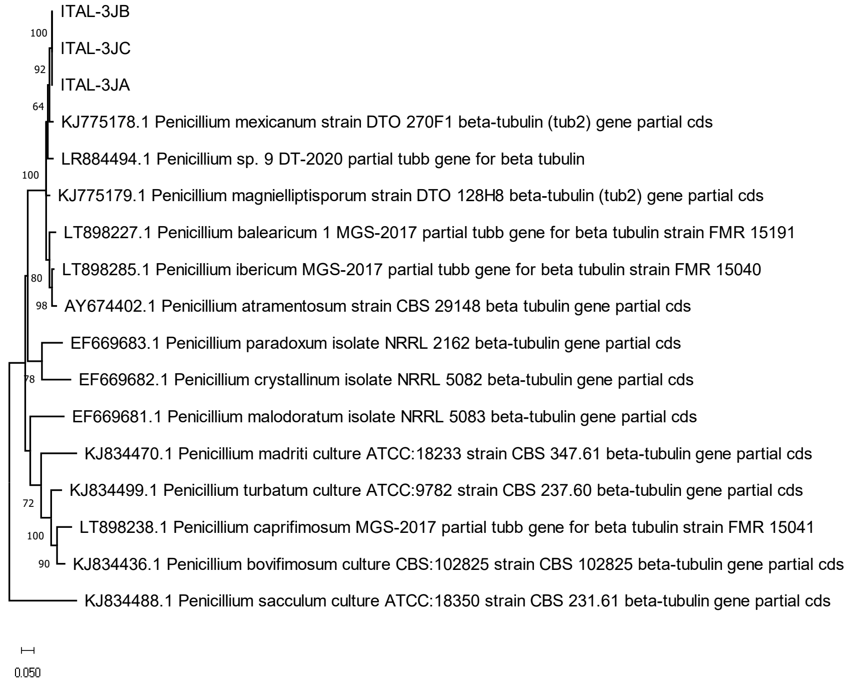


**Figure S2C.** Maximum-likelihood tree (T93+G+I) of *Penicillium* series: *Paradoxa, Atramentosa* and *Turbata* based on *BenA* data sequences. Only bootstraps ≥ 60% are shown. *Penicillium sacculum* is the outgroup.

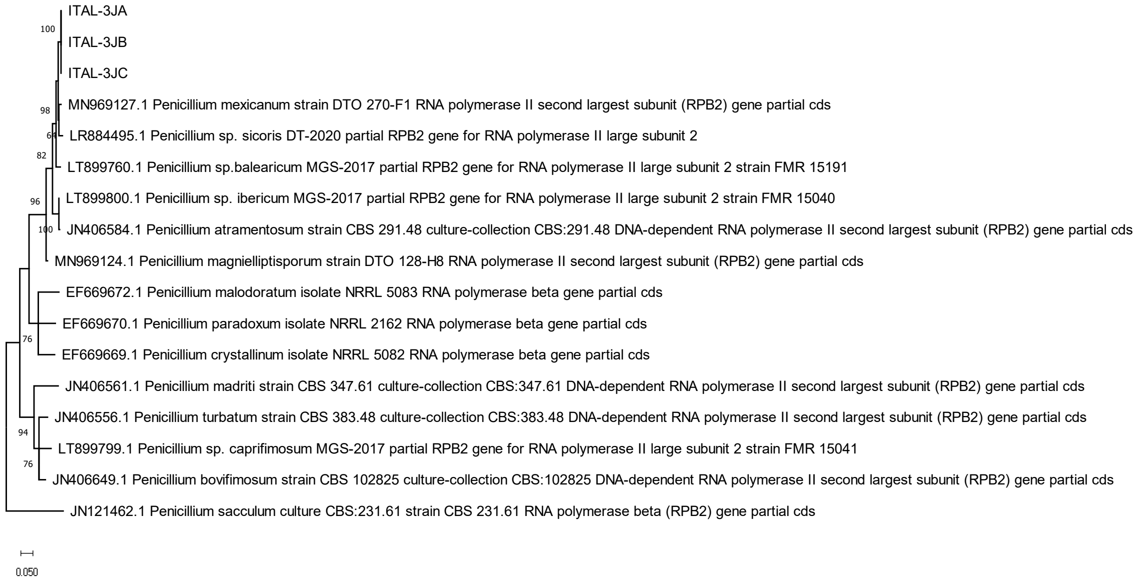


**Figure S2D.** Maximum-likelihood tree (T92+G+I) of *Penicillium* series: *Paradoxa, Atramentosa* and *Turbata* based on *RPBII* data sequences. Only bootstraps ≥ 60% are shown. *Penicillium sacculum* is the outgroup.

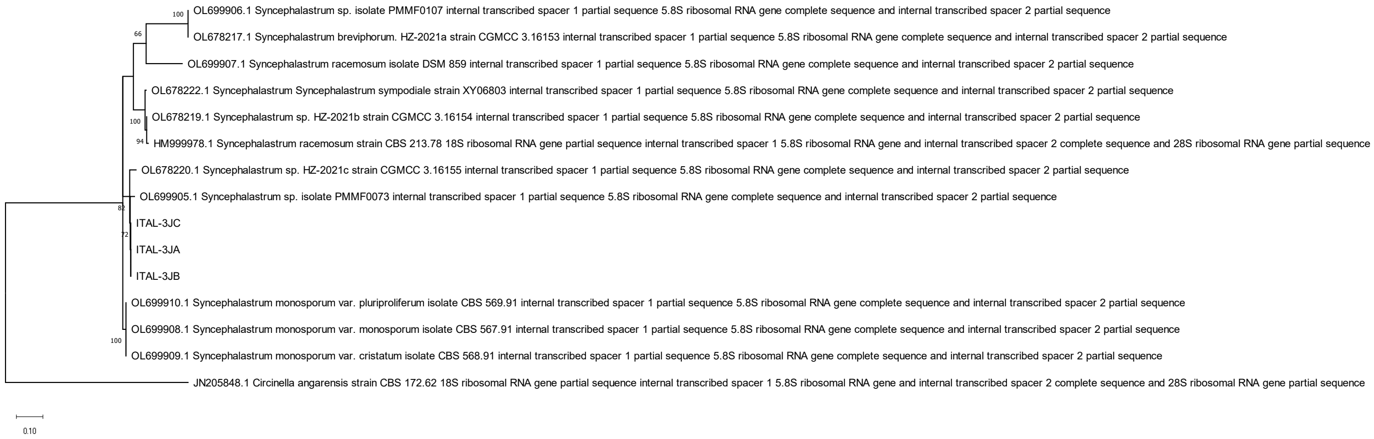


**Figure S2E**. Maximum-likelihood tree (HKY+G) of *Syncephalastrum* genus based on *ITS* data sequences. Only bootstraps ≥ 60% are shown. *Circinella angarensis* is the outgroup.

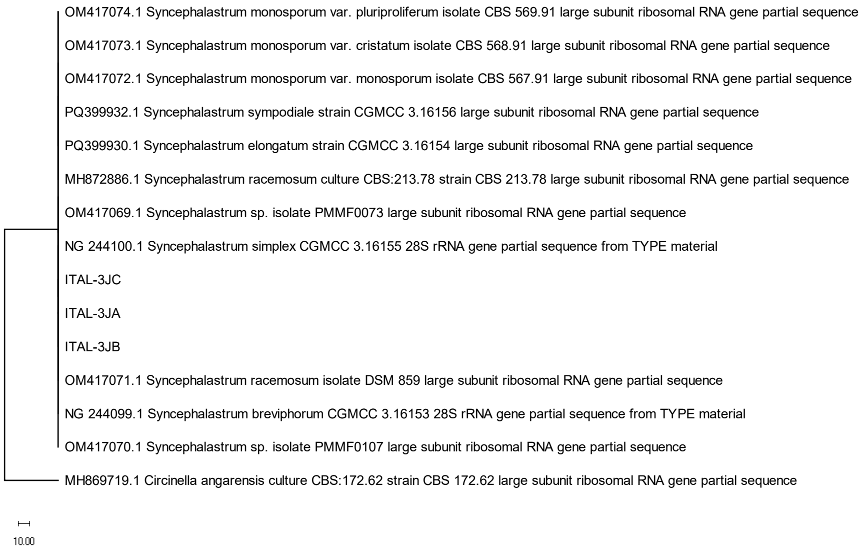


**Figure S2F**. Maximum-likelihood tree (HKY+G) of *Syncephalastrum* genus based on *LSU* data sequences. Only bootstraps ≥ 60% are shown. *Circinella angarensis* is the outgroup.

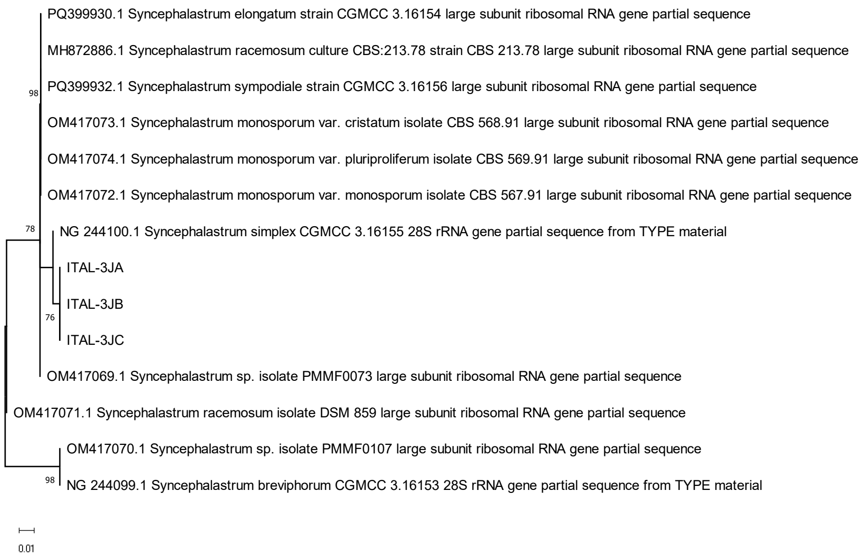


**Figure S2G**. Unrooted Maximum-likelihood tree (HKY+G+I) of *Syncephalastrum* genus based on *LSU* data sequences. Only bootstraps ≥ 60% are shown.
